# Diffuse large B‐cell lymphoma versus Burkitt lymphoma with discordant diagnostic cytogenetics: Morphology trumps

**DOI:** 10.1002/jha2.318

**Published:** 2021-10-26

**Authors:** Kevin E. Shopsowitz, Tracy Tucker, Mohammad Bahmanyar, Pedro Farinha

**Affiliations:** ^1^ Department of Pathology and Laboratory Medicine University of British Columbia Vancouver Canada; ^2^ British Columbia Cancer Agency Vancouver Canada; ^3^ Department of Pathology and Laboratory Medicine Providence Health Care Vancouver Canada

**Keywords:** cytogenetics, lymphomas, morphology

## 1 IMAGE DISCUSSION

**FIGURE 1:**
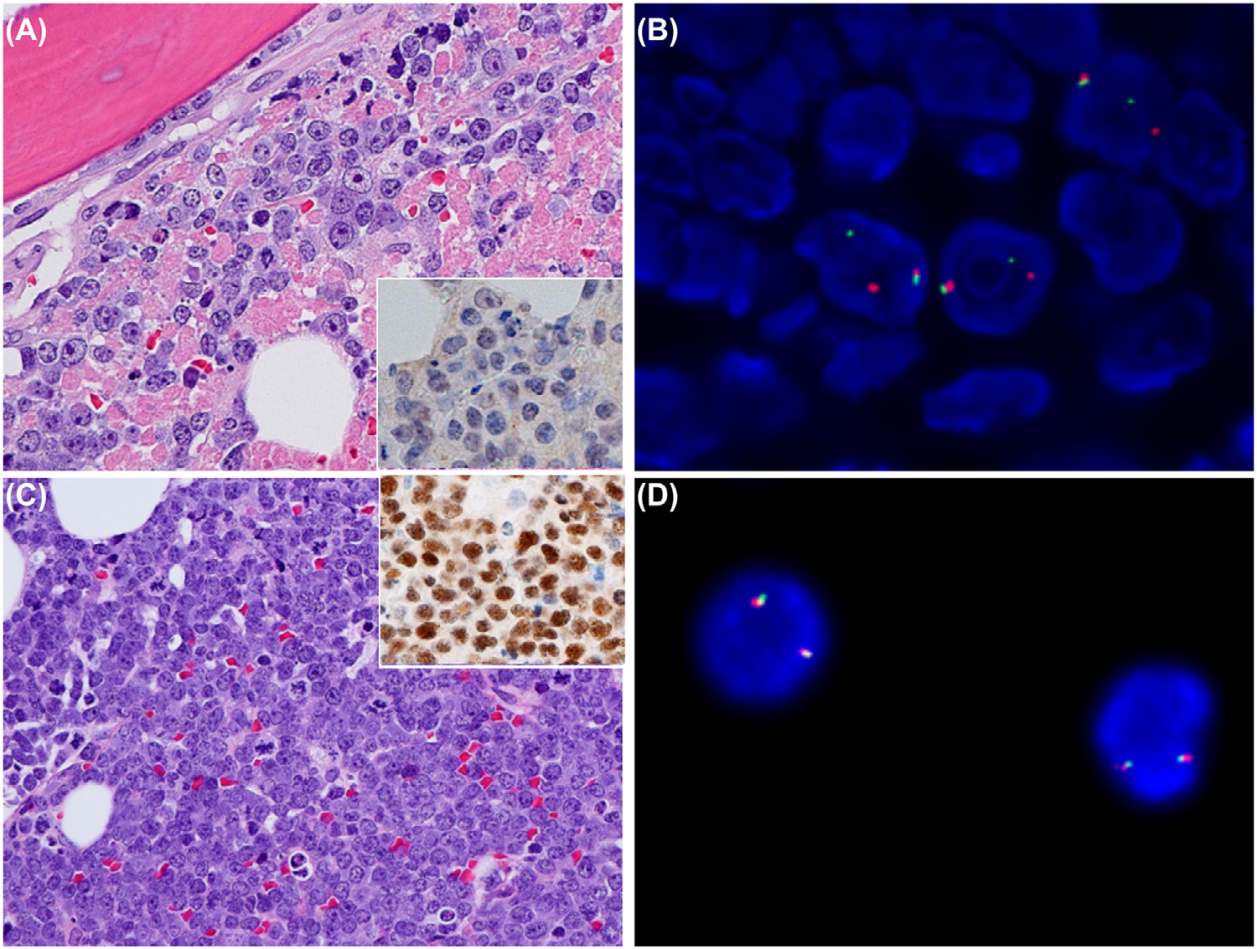
Comparison of bone marrow morphology and FISH for case 1 (DLBCL) and case 2 (BL). (A) Bone marrow biopsy from case 1 showing pleomorphic medium to large cells with prominent nucleoli and necrosis (H&E,40× objective); IHC staining for MYC is negative (inset). (B) FISH for case 1 using a *MYC* break apart probe is positive for a rearrangement, discordant with the IHC result. (C) Bone marrow biopsy from case 2 showing monomorphic small‐ to medium‐sized cells with starry sky appearance and frequent mitoses (H&E,40× objective); IHC staining for MYC is strongly positive (inset). (D) FISH for case 2 using a *MYC* break‐apart probe is negative for a rearrangement, again discordant with the IHC result

*Case 1*: A 35‐year‐old male with newly diagnosed HIV infection presented with diffuse lymphadenopathy, pancytopenia (hemoglobin 97 g/L, platelets 30 × 10^9^/L, neutrophils 1.9 × 10^9^/L), and a leukoerythroblastic blood film. Bone marrow and lymph node biopsies both showed a diffuse infiltrate of variable medium to large lymphoid cells with a starry sky appearance, prominent nucleoli, and frequent mitoses with large areas of necrosis seen in the marrow (Figure 1A, 40× objective). The infiltrating cells had a germinal center B‐cell (GCB) phenotype (CD20/CD10/BCL6 positive), were BCL2 negative, and showed a variable proliferation index of 70%–95%. Although MYC was uniformly negative by immunohistochemistry (IHC; Figure 1A inset, 40× objective), a *MYC* translocation was identified by fluorescence in situ hybridization (FISH; Figure 1B), highly suggestive of a false negative IHC result. As BCL2/BCL6 rearrangements by FISH were negative, Burkitt lymphoma (BL) was considered. However, diffuse large B‐cell lymphoma (DLBCL) was favored given the morphology and variable proliferation index.

*Case 2*: A 71‐year‐old male presented with easy bruising and night sweats. He was found to have severe thrombocytopenia (platelets = 5 × 10^9^/L) and a leukoerythroblastic blood film containing abnormal circulating lymphoid cells with vacuolated basophilic cytoplasm. The bone marrow biopsy showed an atypical B‐cell infiltrate with focal starry‐sky appearance, but in this case they were monomorphic small to medium‐sized cells, mostly with multiple small nucleoli (Figure 1C, 40× objective). The abnormal B‐cells again had a GCB phenotype; however, MYC IHC was strongly positive (Figure 1C inset, 40× objective) and Ki‐67 was uniformly >95%. BL was suspected, but surprisingly FISH was negative for a *MYC* rearrangement (Figure 1D) or 11q abnormality. Despite these negative results, BL was ultimately diagnosed based on the combination of morphology, immunophenotype, uniformly high proliferation index, and MYC protein overexpression, strongly suggestive of a cryptic *MYC* rearrangement.

DLBCL and BL are aggressive B‐cell lymphomas that can show overlapping immunophenotypic and cytogenetic features. Although an isolated *MYC* translocation is a hallmark of BL, this finding can also be found in other aggressive B‐cell lymphomas, such as DLBCL, while cryptic *MYC* rearrangements—negative by conventional FISH analysis—have been reported in BL. These cryptic rearrangements, along with other well‐documented mechanisms such as mutations or polymorphisms, can result in apparently discordant results between MYC IHC and *MYC* FISH. The two cases presented here highlight how morphology, immunophenotype, and cytogenetics must be considered together to distinguish DLBCL from BL using the current conventional diagnostic criteria (WHO 2016). For these cases, morphology was crucial leading to opposite final diagnoses than would be expected based solely on cytogenetics.

## AUTHOR CONTRIBUTIONS

Kevin E. Shopsowitz compiled the figure and prepared the case discussion. Tracy Tucker analyzed the FISH for the cases and provided the images. Mohammad Bahmanyar and Pedro Farinha were senior pathologists involved in the cases, collected images, and contributed to manuscript preparation.

## CONFLICT OF INTEREST

The authors declare no conflict of interest.

